# Signal suppression makes search less effortful

**DOI:** 10.3758/s13415-025-01345-6

**Published:** 2025-09-18

**Authors:** Brad T. Stilwell, Brian A. Anderson

**Affiliations:** 1 Texas A&M University, College Station, TX USA; 2 Department of Psychology, Wake Forest University, Winston-Salem, NC USA

**Keywords:** Visual attention, Attentional capture, Suppression, Salience, Effort

## Abstract

Physically salient stimuli compete for attention but can be suppressed under certain conditions. Highly salient distractors can be suppressed *more* efficiently than less salient ones. However, the implications for the suppression of salient-but-irrelevant signals on the subjective effort of searching are unclear. On one hand, the neural processes involved in signal suppression may themselves be effortful to engage. On the other hand, the facilitation of search that results from reduced competition from the distractor in the visual system may render the act of searching less mentally effortful. Using a recently developed technique of relating physical effort to the putative mental effort required by different search conditions, we assessed whether observers were more motivated to exert physical effort to avoid the demands of ignoring high- or low-salience distractors. We replicated greater suppression for high- than low-salience distractors and showed that participants exerted more physical effort in exchange for search displays containing the high-salience distractors. However, in a situation where high- and low-salience distractors captured attention equally, participants no longer exhibited this preference. Our results suggest that observers prefer the conditions in which they search most efficiently, even when those conditions involve stronger recruitment of suppressive mechanisms of distractor processing.

Humans often design objects that convey useful information by rendering them in ways that help them to “stick out” from neighboring items (e.g., a high-contrast yellow wet floor sign or a bright flashing traffic signal). By making these items *perceptually salient*, the hope is that they will automatically capture people’s attention, signaling the pertinent information. The question of whether salient items automatically capture attention has been heatedly debated for decades (Luck et al., 2021). We argue that perceptually salient stimuli can, under certain conditions, be suppressed by the attention system and that more salient stimuli can be more robustly suppressed. We then leverage this relationship between salience and suppression to examine the relationship between signal suppression and the effort required for attentional control.

## Signal suppression and the attention capture debate

According to *stimulus-driven* accounts of attentional control, the most salient item will automatically capture attention irrespective of one’s goals (Jonides & Yantis, 1988; Theeuwes, 1992, 1994, 2010). Evidence for these accounts often comes from studies using the *additional singleton paradigm* in which participants search for a uniquely shaped target among homogenously shaped nontarget items (e.g., a diamond target among circles nontargets). Critically, one of the nontarget items can be rendered in a unique color, forming a *color singleton distractor*. The canonical finding is that participants are slower to respond to the target when the color singleton distractor is present than when it is absent, a *singleton-presence cost* (Theeuwes, 1992). Likewise, under such conditions, the salient distractor evokes elevated signals within the visual and attentional systems of the brain (Adam & Serences, 2021; Corbetta & Shulman, 2002; Serences et al., 2005). This slowing of responses to the target and robust distractor processing in the brain are argued to result from attentional capture by the singleton distractor before reallocating attention toward the target. However, the salient color singleton seems to only capture attention when participants adopt a control setting to search for singletons more generally, a *singleton-detection* mode (Pashler, 1988). Searching for the uniquely shaped item causes all items with unique properties, such as color singleton distractors, to compete for attentional priority. If instead, participants search for a specific shape (e.g., a circle) among heterogeneously shaped nontargets, participants tend to adopt a search strategy to find the specific shape (Bacon & Egeth, 1994; Lamy & Egeth, 2003). This *feature-search* mode causes the salient color singleton distractor to no longer capture attention, which is consistent with *goal-driven* accounts that propose the goals of the observer or the *attentional set* afforded by the task drive attentional priority (Bacon & Egeth, 1994; Folk & Remington, 2010; Folk et al., 1992; Irons et al., 2012). Correspondingly, under conditions of feature search, salient distractors evoke significantly more robust activity within the visual and attentional systems of the brain when they possess a task-relevant feature that is prioritized by the attentional set (Andersen et al., 2011; Fahrenfort et al., 2017; Grubert & Eimer, 2018; Martinez-Trujillo & Treue, 2004; Serences & Yantis, 2006, 2007; Serences et al., 2005); that is, the attentional processing of a distractor is *contingent* upon its relationship to the attentional set.

To resolve these opposing theoretical perspectives, the *signal suppression hypothesis* was proposed as a hybrid account appealing to aspects of both positions (Gaspelin et al., in press). According to the signal suppression hypothesis, salient stimuli will automatically compete for attentional priority, but such stimuli can be suppressed consistent with the observer’s top-down goals to prevent the capture of attention (Gaspelin & Luck, 2018c, 2019; Sawaki & Luck, 2010). The signal suppression hypothesis has garnered much recent support across converging methods using psychophysical approaches, such as the capture-probe paradigm (Chang & Egeth, 2019; Drisdelle et al., 2025; Gaspelin et al., 2015; Ma & Abrams, 2023a; Stilwell & Gaspelin, 2021), eye tracking (Adams & Gaspelin, 2024; Adams et al., 2022; Gaspelin et al., 2017; Hamblin-Frohman et al., 2022; Zhang & Gaspelin, 2024; Zhang et al., 2025b), electrophysiological indices (Burra & Kerzel, 2014; Drisdelle & Eimer, 2021, 2023; Feldmann-Wüstefeld et al., 2020; Gaspelin & Luck, 2018a; Gaspelin et al., 2023; Sawaki et al., 2012; Tam et al., 2022), and single-unit recordings in nonhuman primates (Cosman et al., 2018; Sapountzis et al., 2025).

Despite strong evidence in favor of the signal suppression hypothesis, the account has received criticism for using stimuli that might not have been perceptually salient enough to warrant suppression (Theeuwes in Luck et al., 2021; Theeuwes, 2021; Wang & Theeuwes, 2020). However, this *low-salience* account has been refuted by several recent studies suggesting that highly salient distractors can be suppressed (Drisdelle & Eimer, 2023; Ramgir & Lamy, 2023; Stilwell & Gaspelin, 2021; Stilwell et al., 2022, 2023; Gaspelin et al.,  in press). In fact, as described in the following section, there is evidence that more salient stimuli can lead to greater suppression.

## Perceived salience and its role in attentional suppression

Recent attempts have been made to quantify perceived salience (Chang et al., 2021; Stilwell & Gaspelin, 2021; Stilwell et al., 2023). One approach used a combination of computational models and psychophysical approaches to assess perceived salience (Chang et al., 2021; Stilwell & Gaspelin, 2021; Stilwell et al., 2022), and several authors have employed intuitive manipulations of perceived salience (e.g., via increasing the set size of the display or increasing the color contrast between search items), with the results consistently demonstrating that highly salient singletons can be suppressed (Drisdelle & Eimer, 2023; Ramgir & Lamy, 2023; Stilwell & Gaspelin, 2021; Stilwell et al., 2022, 2024; Zhang et al., 2025a). However, without an agreed-upon tool to assess perceived salience, claims concerning the role of salience in attentional capture are difficult to test.

Recently, Stilwell et al. (2023) developed a psychophysical approach which can be used to assess the perceived salience of stimuli (see also Stilwell et al., 2024). In their study, Stilwell et al. (2023) used an *oddball detection task* to measure the perceived salience of a manipulation of salience via color contrast (e.g., red among blue items [high color contrast] vs. teal among blue items [low color contrast]) by briefly presenting displays containing a color singleton and manipulating the duration needed to reliably detect the colors based on their contrast. Participants had lower exposure thresholds for detecting the high- than the low-contrast singletons, suggesting they perceived the high-contrast singletons as more salient than the low-contrast ones. Following the oddball detection task, the same participants performed a visual search task using the same displays containing the salient color singletons, which then served as distractors to be ignored. Using eye tracking measures, the authors found that oculomotor suppression—fewer first saccades directed at the singleton distractor than nonsingleton distractor items—was robust for both the high- and low-salience distractors. Importantly, the magnitude of oculomotor suppression was greater for the high- than the low-salience distractors. This suggests that rendering the distractors as more salient actually led to greater suppression, not greater capture (Zhang & Gaspelin, 2024). The authors argued that the high-salience distractors were *easier* to suppress than the low-salience distractors. However, this raises an important question: What does it mean for suppression to be *easier* for higher salience distractors? Does greater suppression require greater cognitive control, which is effortful, or does the resulting reduction in competition from the salient distractor make the task of attending to the target easier?

### Physical effort and search efficiency

If observers can suppress highly salient color singleton distractors more efficiently than less salient distractors, it seems plausible that this difference should lead to differences in perceived difficulty of the visual search task. In other words, if suppressing the high-salience distractors serves a benefit to visual information processing, participants should be more inclined to perform these searches over searches containing less salient distractors, if given the choice. Humans typically make choices that serve to conserve energy and prefer tasks that require minimal effort (Klein-Flügge et al., 2016; Kurniawan et al., 2010; Prévost et al., 2010). For example, if given the choice to squeeze a hand dynamometer in exchange for less demanding visual search tasks, participants are more willing to exert physical effort to make their search task easier as a function of the putative difficulty of the search task (Anderson & Lee, 2023; Anderson et al., 2025; Anderson, 2024b; Lee et al., 2024). Lee et al. (2024) had participants search displays that promoted either singleton-detection or feature-search mode in the additional singleton paradigm. Participants were reliably faster and more accurate in responding to the target using feature-search mode than they were using singleton-detection mode. Furthermore, when given the option to exert physical effort in exchange for choosing the type of displays afforded by each search mode, participants were reliably more willing to exert physical effort via squeezing the hand dynamometer to switch the task from singleton-detection mode to feature-search mode than vice versa. These results, and others (Anderson, 2024b), suggest that participants are more willing to “trade-in” physical effort in exchange for tasks that promote greater visual search efficiency (Anderson et al., 2025).

A recent theoretical account proposed that involuntary mechanisms of attentional control often serve to minimize the need for effortful information processing (Anderson, 2021a, 2024c). According to the model, attentional priority is accumulated through the combined activation of multiple inputs (e.g., statistical learning, valence associations, and current task-relevance), all of which interact with visual salience and memory systems via tonic (i.e., sustained neural activity such as the observer’s control settings) and phasic (i.e., transient modulations of neural signals based on the tonic activity) control signals. Under this type of framework, it seems plausible that distractor suppression, for example using a signal suppression mechanism, would use tonic and phasic changes in neural activity to attenuate the salient signals generated by the color singleton distractors in perceptual systems, thereby reducing perceptual competition from the distractors. Suppressing the distracting signals should therefore lead to more efficient, and potentially, less cognitively demanding visual search.

## Current study

If observers are more willing to exert physical effort to make their search task more efficient (see Anderson et al., 2025, for a review) and rendering distractors more perceptually salient leads to more efficient information processing via suppressive mechanisms, it stands to reason that participants should be motivated to trade physical effort in exchange for task conditions that promote such suppression (i.e., conditions where the distractor is highly salient). Improved distractor suppression should make the search task *less* effortful because ruling out a salient distracting item as a potential target item should reduce the number of items needed to be searched. Alternatively, the ability to suppress more salient distractors might be *more* effortful by recruiting more resources from areas, such as prefrontal cortex (for review see Sarter et al., 2006). A more salient signal might require a greater level of activity within attentional control modules in the brain to suppress it, which in turn might be more effortful to engage and metabolically demanding (Braver et al., 2009; Engelmann & Pessoa, 2007; Jimura et al., 2010; Locke & Braver, 2008; Pessoa & Engelmann, 2010; Pessoa et al., 2003). Increasing the salience of the singleton distractor leads to more efficient suppression of that distractor, but the relationship between such suppression and the perceived effort of searching remains to be tested.

In the current study, we manipulated the salience of the singleton distractor via color contrast (Stilwell et al., 2023) and then tested whether participants would exert physical effort to change the salience of the distractors in an upcoming block of trials. We hypothesized that if participants show a difference in distractor suppression between levels of salience, then they should be more willing to exert physical effort to select the level of salience for which search was less effortful. In experiment 1, we adopted displays to promote feature-search mode which, in turn, should promote suppression of the high-salience singleton distractor. To replicate previous findings, we expected to observe greater oculomotor suppression for the high- than low-salience distractors (Stilwell et al., 2023; Zhang & Gaspelin, 2024). Importantly, we hypothesized that more robust suppression of the salient distractor would reduce the effort required to perform the search task, resulting in participants being more willing to exert physical effort in exchange for the opportunity to search among more highly salient distractors. If instead participants find the more salient distractor to be more effortful to suppress, despite their improvement in visual search efficiency, they should be more willing to exert physical effort in exchange for the opportunity to search among less salient distractors. A third possibility is that participants might also show no preference for either condition of salience given that people seem to have a limited conscious access to how they direct their attention in these types of visual search tasks (Adams & Gaspelin, 2021; Anderson & Mrkonja, 2022; Horowitz & Wolfe, 1998; Võ et al., 2016) or potentially offsetting influences of benefits due to increased search efficiency and costs due to more effortful engagement of suppressive mechanisms.

## Experiment 1

Experiment 1 contained two phases. In the first phase of the experiment, participants performed a visual search task in which they searched for a specific shape target among heterogeneously shaped nontargets, which is known to lead to suppression of a salient color singleton distractor (Stilwell et al., 2023). Participants gained experience with high- and low-salience distractors, which provided an opportunity to become familiarized with the demands of searching amidst each. In the second phase of the experiment, participants had the option to squeeze a hand dynamometer in exchange for changing the color of the singleton distractor prior to each search block (Anderson et al., 2025), making the distractor more or less salient.

### Method

#### Participants

An a priori sample size of 32 participants was determined based upon a previous study (Stilwell et al., 2023). Part 1 contains a direct replication of the attentional capture task from Stilwell et al., (2023). We conducted a power analysis based on the effect size of the difference in oculomotor suppression effects between the high- and low-salience conditions from that experiment (*d*_*z*_ = 1.05), and 26 participants would be needed to obtain 99.9% power. We collected 32 participants to ensure counterbalancing of the relevant conditions (see below). The participants were students from Texas A&M University who participated for course credit after obtaining written informed consent (17 women, 15 men; mean age = 19.3 years). All participants were English-speaking and reported normal or corrected-to-normal visual acuity and normal color vision. All procedures were approved by the Texas A&M University Institutional Review Board and were conducted in accordance with the principles expressed in the Declaration of Helinski.

#### Apparatus

Stimuli were presented on a Dell P2717H monitor using MATLAB software and PsychToolbox (Kleiner et al., 2007) via a Dell OptiPlex 7040 computer. Responses were recorded via a 5-button, MilliKey MH-5 response box. Participants were seated at a distance of approximately 70 cm in a dimly lit room.

#### Search stimuli

**Fig. 1:**
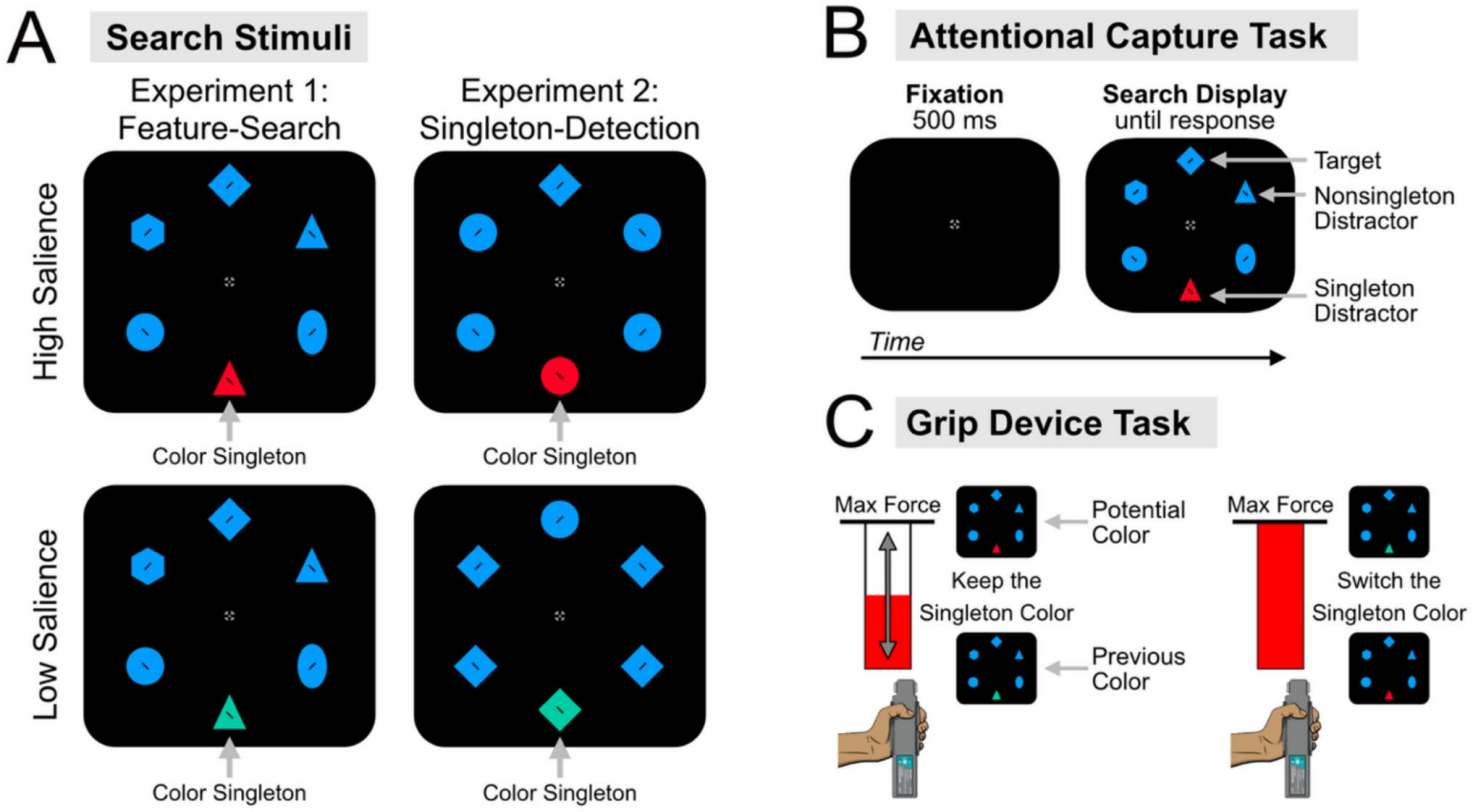
Stimuli and tasks for experiments 1 and 2. (**A**) Participants searched for either a specific shape target (i.e., diamond or circle) among heterogeneously shaped nontargets in experiment 1 or a uniquely shaped target (i.e., the diamond among circles or circle among diamonds) among homogenously shaped nontargets in experiment 2. For both experiments, participants attempted to ignore the color singleton distractor when present. The color contrast of the singleton distractor was varied by experimental half to form two conditions of singleton salience: high and low salience. (**B**) For both phases of the experiment, participants responded to the orientation of the line segment (either left- or right-tilted) contained within the target. (**C**) In phase 2 of the experiment, participants squeezed a hand dynamometer prior to each block of trials to choose which color the singleton distractor was rendered in during the next block. If participants squeezed to their maximum calibrated force, they switched the color of the distractor (high or low salience) from the previous block. If participants squeezed any amount below their calibrated max, they kept the same distractor color

As depicted in Fig. 1A, all trials consisted of a search display containing six colored shapes arranged equidistant around an imaginary notational circle 6.83° from the center of the screen. The fixation cross, which consisted of a gray (50 cd/m^2^, *x* = .301, *y* = .346) circle (0.81° visual angle diameter) cross-sectioned by two black rectangles (each 0.81° by 0.20°), with a gray dot in the center (0.20° diameter), was presented in the center of the screen and remained visible throughout the experiment. Each shape filled up an imaginary rectangle with dimensions subtending approximately 2.73° by 2.73° visual angle. The displays always contained both a circle and a diamond, one of which was the target, counterbalanced across participants. The remaining nontargets consisted of at least one hexagon, one oval, and one triangle—of which, one was randomly duplicated on each trial. To encourage eye movements, each shape contained a tiny black line segment (length of 0.3° and a width of 0.1°) that was tilted 45° to the left or right.

The colors of the shapes were either red (50.0 cd/m^2^, *x* = .646, *y* = .324), blue (50.0 cd/m^2^, *x* = .189, *y* = .252), pink (50.0 cd/m^2^, *x* = .610, *y* = .305), or teal (50.0 cd/m^2^, *x* = .215, *y* = .368). These colors were predetermined by selecting portions of a CIE color space (*L* = 50, radius = 90) and then adjusted to be photometrically isoluminant on the display monitors. On singleton-absent trials, all items were rendered in the same color (e.g., blue). This color was counterbalanced across participants and remained consistent throughout the experiment. On singleton-present trials, one nontarget shape was rendered in a unique color, creating a color singleton distractor. The color contrast between the singleton distractor and the other display items was manipulated (Fig. 1A) to form two conditions of high-salience, which were separated by 180° in color (e.g., red and blue) or low-salience, which were separated by 27° in color (e.g., teal and blue). These colors have been empirically verified using an oddball detection task (inspired by Greene & Oliva, 2009; and suggested by Theeuwes in Wöstmann et al., 2022) to produce differences in perceived salience (Stilwell et al., 2023, 2024; Zhang & Gaspelin, 2024; Zhang et al., 2025a).^1^

#### Design and procedure

Each participant was assigned to search for a target defined by both shape (i.e., a diamond or circle) and color (i.e., red, blue, teal, or pink), both of which remained consistent throughout both phases of the task. A color singleton distractor was present on 75% of trials and appeared at a random location with the constraint that it could not appear at the target location. Participants were instructed to ignore the color singleton distractor when present. Participants reported the orientation of the line segment (i.e., left- or right-tilt) contained within the target shape via a speeded button press. If the target line was right-tilted, participants pressed the right button (bottom right button on the response box) and if the target line was left-tilted, participants pressed the left button (bottom left button on the response box).

To initiate the trial, participants had to maintain central fixation (i.e., within a circular region of 2.28° centered around the middle point of the screen) for 500 ms. If participants could not maintain fixation for 500 ms within a 5,000-ms interval, the trial did not begin and participants were recalibrated. Following successful central fixation, the search display immediately onset and remained on screen until participants made a response or a 3000 ms search timeout was reached. If participants responded inaccurately, a screen with the feedback “Incorrect!” appeared after their response. If a participant took longer than 3000 ms to respond, a screen with the feedback “Too Slow” appeared. Both feedback screens were accompanied by a 200-Hz beep that lasted 300 ms. Following either failure to respond correctly, a 1,500-ms pause was inserted to allow participants recovery time before the next trial. At the end of each block, participants were shown a summary screen depicting their average RT and accuracy and were encouraged to maintain sufficiently fast and accurate responses: Participants were warned if their mean RT exceeded 1,500 ms or their mean accuracy dipped below 85% correct.

After giving consent, prior to beginning the experimental tasks, participants were calibrated using a 9-point calibration procedure via an EyeLink 1000 Plus eye tracker. Following successful calibration, participants calibrated the Vernier Hand Dynamometer. To calibrate the grip device, participants were told to pick up the grip device with their dominant hand. The following series of text prompts each appeared for 1000 ms: “Ready…” (1000 ms), “Set…” (1,000 ms), “SQUEEZE!” (2,000 ms). While the “SQUEEZE!” text was presented, participants were told to squeeze and sustain their maximum grip force. Participants were then told to relax for 500 ms before the cycle was repeated. The median value of grip force (in Newtons) over the calibration duration was used as the maximum grip strength for each participant (see below). Following calibration of both devices, participants were instructed on the attentional capture task.

##### Phase 1: Attentional capture task

 The first phase of the experiment was directly modeled after the attentional capture task from Stilwell et al. (2023) in which participants searched for a shape-defined target while ignoring a color singleton distractor. The salience of the color singleton was blocked to create two halves, one for each level of singleton salience (high- vs. low-salience). Each participant was assigned either the high- or low-salience singleton for the first half and then the opposite color for the second half. The order of halves was counterbalanced across participants. Participants began the experiment with one block of practice containing 48 singleton-absent trials to familiarize them with the task and button mappings. Following practice, each half (i.e., level of singleton salience) contained three blocks consisting of 48 trials each, totaling six experimental blocks of 288 trials. Of these 288 trials, the singleton distractor was presented 75% of the time resulting in 216 singleton-present trials (108 trials for each level of salience) and 72 singleton-absent trials.

##### Phase 2: Grip task

During the second phase of the experiment, immediately following the first phase, the same participants continued with the same attentional capture task from phase 1 with the following exceptions. The colors (i.e., the target/nonsingleton and singleton distractor) remained consistent throughout the experiment. This was done to both align with the extant literature in which suppression is more robust when the to-be-suppressed feature remains constant (Gaspelin & Luck, 2018c; Stilwell et al., 2023; Zhang & Gaspelin, 2024) than when it changes unpredictably (Adam & Serences, 2021) and to encourage participants to treat the search task in both phases as similar as possible with the only Change being the grip manipulation introduced in phase 2.^2^ Prior to each block, participants were informed that they would utilize the grip device to select which distractor color they wanted for the entirety of the next block of trials. Participants were shown a meter that, depending on how hard they squeezed, would fill with a red bar (Fig. 1C) to a degree that was calibrated to their maximum grip strength. The fill bar consisted of five tick marks representing between 0 and 100% in 20% increments, calibrated to each participant’s maximum grip strength (represented by a green line that extended horizontally past the vertical edges of the fill bar in both directions). Participants were told that they had the option to exert physical effort via the grip device to either switch or keep the singleton distractor color during the next block of search trials. To the right of the fill bar, participants were shown example stimulus arrays which were lined up with the bottom and top of the fill bar. The bottom array represented the color of the singleton distractor during the previous block of trials and the top array represented the potential color of the singleton distractor if participants chose to max out their grip strength (i.e., fill the meter to the green line at the top). If participants squeezed at or above their calibrated maximal force, this would trigger a switch of the singleton’s color from the prior block to the opposite color for the next search block. If participants squeezed the device at any percentage below their calibrated maximum (including not squeezing at all), this would cause the singleton color to remain the same as the prior block. In other words, participants did not need to exert *any* force throughout the entire phase if they did not want to switch the distractor color from the previous block. If a participant did not grip the device at all (or if they squeezed below their calibrated maximum) the distractor color remained the same as in the previous block, which could either be the color initially assigned in the first block (counterbalanced across participants) or the color that resulted from the last time the participant chose to switch via exerting their calibrated maximum force. Participants were explicitly told they had no obligation to squeeze the device and that they could squeeze the device as much or little as they wanted to. Whenever participants were satisfied with their choice, they could press either button box response key, with or without simultaneously gripping the device, to lock in their choice of color. However, squeezing the device to the max immediately locked in the choice to switch the color singleton. Regardless of the choice, participants were then notified of their choice with text that appeared in the center of the screen stating either: “You chose to SWITCH the colors” or “You chose to KEEP the colors.” After their choice, participants searched through displays in a block of 48 trials of the attentional capture task from phase 1 with the singleton distractor color they chose.

For phase 2, there was a total of six experimental blocks and one practice block each containing 48 trials. The choice of singleton color for the first block of trials (i.e., practice) was fixed and counterbalanced across participants: Half of participants were assigned the high- and the other half of participants were assigned the low-salience singleton distractors for the first block. Following practice, the choice via the grip device determined the color of the singleton distractor within of each of the six experimental blocks; therefore, participants each had six opportunities to choose which singleton distractor color they preferred. All other aspects of phase 2 were identical to phase 1.

#### Data analysis

The first block of each phase was excluded as practice. Trials with an RT less than 200 ms or greater than 2,000 ms (1.1% of trials in phase 1, 0.7% of trials in phase 2) were removed from analysis. Furthermore, trials with abnormal saccadic latencies less than 50 ms or greater than 1,000 ms (1.3% of trials in phase 1, 1.0% of trials in phase 2) and trials where participants did not move their eyes from central fixation (0.2% of trials in phase 1, 0.8% of trials in phase 2) were removed. Additionally, trials containing incorrect responses (2.4% of trials in phase 1, 2.3% of trials in phase 2) were excluded from all analyses. These trimming procedures resulted in a total of 4.7% and 3.9% of trials excluded from phases 1 and 2, respectively.

First eye movements were measured to evaluate attentional suppression of the color singleton distractor by subtracting the percentage of first eye movements directed toward the singleton distractor from the average nonsingleton distractor. This *oculomotor suppression effect* reflects below-baseline attentional suppression of the color singleton distractor (Adams & Gaspelin, 2024; Gaspelin et al., 2017; Stilwell et al., 2023). Saccades were defined by a minimum acceleration of 9,500°/sec^2^ and a minimal eye velocity threshold of 30° per second. First saccade destinations were defined using an annulus around the search array, with an inner radius of 1.4° from fixation and an outer radius of 7.1° from fixation. The first eye movement landing within the annulus was classified as the first saccade and the nearest search item was then selected as the first saccade destination. This effectively creates wedge-shaped interest areas around each search item (Leonard & Luck, 2011). Saccadic latency was measured as the start time of the first saccade that landed within the annulus. For all within-subject *t* tests, Cohen’s *d* (one-sample) or *d*_*z*_ (paired-samples) were used to measure effect size (Lakens, 2013).

### Results

#### Phase 1: Attentional capture task

##### **Manual responses**

Manual RTs were faster on singleton-present trials than singleton-absent trials (a singleton-presence benefit) for both conditions of salience, see Table 1. One-sample *t* tests indicated that the singleton-presence benefits for both the high-salience (23 ms), *t*(31) = 3.43, *p* = .002, *d* = 0.61, and the low-salience distractors (25 ms), *t*(31) = 2.88, *p* = .007, *d* = 0.51, were statistically significant. However, a paired-samples *t* test indicated that the singleton-presence benefits were not significantly different between conditions of salience, *t*(31) = 0.21, *p* = .84, *d*_*z*_ = 0.04, suggesting that both levels of salience were inhibited.^3^ Mean error rates were low and showed neither singleton-presence benefits nor costs (*p*’s > .26). Together, these results suggest that participants were able to inhibit the salient color singleton distractors, regardless of their levels of salience.

**Table 1:** Experiment 1: Mean response time (in ms) and mean error rate (in %) by singleton presence and singleton salience

Mean RTs (ms)				
	Phase 1: Attentional Capture Task		Phase 2: Grip Task	
*Singleton Salience*	Absent	Present	Absent	Present
Low	970 (159)	945 (152)	860 (105)	854 (96)
High	956 (138)	934 (140)	870 (123)	861 (128)
**Mean error rates (%)**				
	Phase 1: Attentional Capture Task		Phase 2: Grip Task	
*Singleton Salience*	Absent	Present	Absent	Present
Low	2.0 (2.5)	2.2 (1.7)	2.7 (3.8)	3.0 (2.5)
High	2.7 (2.9)	2.4 (1.8)	1.4 (1.7)	2.9 (2.1)

Standard deviations are provided in parentheses

##### **First Saccade destination**

**Fig. 2:**
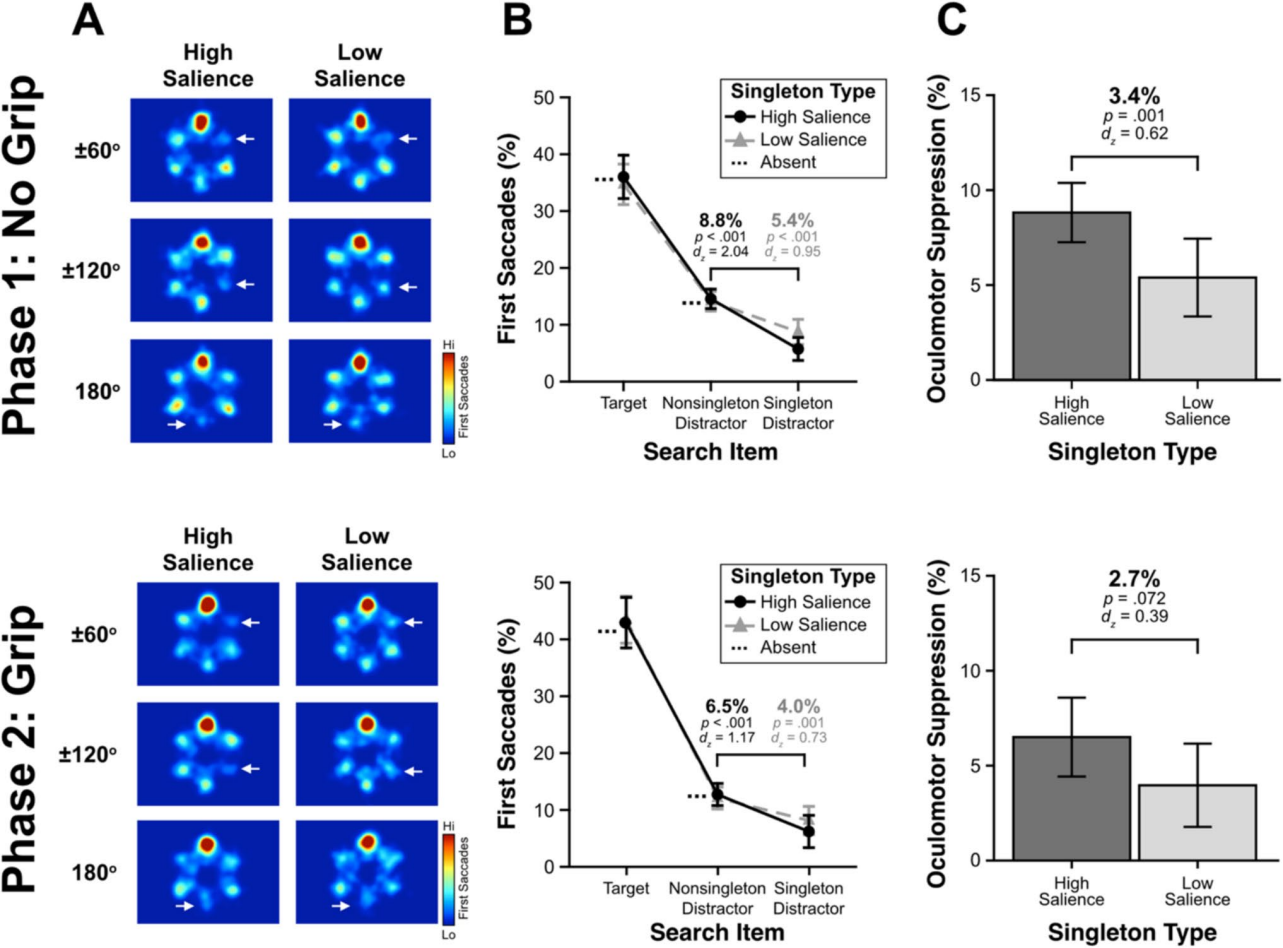
Saccade destination results for experiment 1. Phase 1 results are presented in the top panel and phase 2 results are presented in the bottom panel. (**A**) Heat maps depicting the first saccades as a function of singleton salience and angular distance between the target and the singleton distractor. For ease of viewing, the heat maps were generated and normalized such that the target is represented at the top position and the position of the singleton indicated by white arrows. For simplicity, the angular distances of − 60° and − 120° were flipped on the x-axis and collapsed to form pooled angular distances, the white arrow depicting the singleton distractor’s location. (**B**) The percentage of first saccades to each search item as a function of singleton salience. (**C**) Oculomotor suppression effects (average nonsingleton – singleton distractor) as a function of singleton salience. All error bars represent within-subject, 95% confidence intervals (Cousineau, 2005; Morey, 2008)

The primary aim of phase 1 was to replicate the patterns of greater oculomotor suppression for the high- than low-salience distractors from Stilwell et al. (2023). Figure 2A depicts heat maps for the first saccade as a function of singleton salience and the angular distance between the target and singleton distractor positions. As can be seen, singleton distractors were fixated fewer than any other search item in the display regardless of salience, reflecting oculomotor suppression. This ocuolomotor suppression was stronger for high- than low-salience distractors.

To assess these patterns statistically, we compared the percentage of first saccade destinations as a function of the search item fixated and singleton salience (Fig. 2B). The nonsingleton distractor has been divided by the number of nonsingleton distractors (i.e., four on singleton-present trials, five on singleton-absent trials) to provide a per item estimate of being overtly attended. Oculomotor suppression is defined as the difference between first saccades landing on the singleton distractor and the average nonsingleton distractor. These oculomotor suppression effects are depicted in Fig. 2C, where a positive value indicates the salient distractor was suppressed. The high-salience distractors (8.8%), *t*(31) = 11.51, *p* < .001, *d*_*z*_ = 2.04, and the low-salience distractors (5.4%), *t*(31) = 5.37, *p* < .001, *d*_*z*_ = 0.95, were both clearly suppressed. Importantly, the magnitude of suppression was greater for the high- than low-salience distractors, *t*(31) = 3.5, *p* = .001, *d*_*z*_ = 0.62, which replicates the pattern of greater suppression of more salient distractors (Stilwell et al., 2023). These results established that participants learned to suppress the high-salience distractors more efficiently than the low-salience distractors.

#### Phase 2: Grip task

##### **Manual responses**

The magnitude of singleton-presence benefits from phase 1 were reduced in phase 2. One-sample *t* tests indicated that the singleton-presence benefits for both the high-salience (9 ms), *t*(29) = 1.23, *p* = .23, *d* = 0.22, and the low-salience distractors (6 ms), *t*(25) = 0.65, *p* = .52, *d* = 0.13, were not statistically significant, and there was no difference between the conditions of salience, *t*(23) = 0.67, *p* = .51, *d*_*z*_ = 0.14.^4^ As in phase 1, mean error rates were low overall. However, inconsistent with phase 1, in phase 2, participants committed more errors on singleton-present (2.9%) than singleton-absent (1.4%) trials for the high-salience singleton, a singleton-presence cost (1.5%), *t*(31) = 3.33, *p* = .002, *d*_*z*_ = 0.59. Error rates for low-salience singletons did not result in a singleton-presence cost or benefit (0.2%), *t*(31) = 0.34, *p* = .74, *d*_*z*_ = 0.06. Together, these results suggest that participants in phase 2 were more prone to errors in the presence of the high-salience singleton distractors, but this effect did not appear for mean RTs. Nonetheless, as in phase 1, the primary analyses were focused on the oculomotor suppression effects.

##### **First saccade destination**

The oculomotor suppression effects from phase 1 persisted into phase 2 as evident in Fig. 2 (bottom panel). Comparing oculomotor suppression effects, the high-salience distractors (6.5%), *t*(29) = 6.4, *p* < .001, *d* = 1.17, and the low-salience distractors (4%), *t*(25) = 3.72, *p* = .001, *d* = 0.73, were both clearly suppressed. The magnitude of suppression was numerically greater for the high- than low-salience distractors, *t*(23) = 1.89, *p* = .07, *d*_*z*_ = 0.39, although this difference was not statistically reliable. This lack of a difference between the high- and low-salience oculomotor suppression effects in phase 2, which was evident in phase 1, was driven by a Change in the percentage of first saccades toward the target and the nonsingleton items but not the singleton distractor. In comparing phase 1 and phase 2 (Fig. 2B), we found significantly more target fixations in phase 2 than 1 for both the high-salience (7.4%), *t*(29) = 2.67, *p* = .012, *d*_*z*_ = 0.49, and low-salience (8%) conditions, *t*(25) = 2.67, *p* = .013, *d*_*z*_ = 0.52, but significantly fewer nonsingleton fixations in phase 2 for the high-salience (2%), *t*(29) = 3.45, *p* = .002, *d*_*z*_ = 0.63, and low-salience distractors (2.0%), *t*(25) = 2.68, *p* = .013, *d*_*z*_ = 0.53. Importantly, there was no difference in singleton distractor fixations between the phases for high-salience (0.6%), *t*(29) = 0.42, *p* = .68, *d*_*z*_ = 0.08, or low-salience distractors (0.1%), *t*(25) = 0.11, *p* = .91, *d*_*z*_ = 0.02. These results suggest that participants continued to suppress the singleton distractors regardless of their levels of salience in phase 2.

To ensure that the change in task (i.e., introducting the grip manipulation in phase 2) did not fundamentally alter the search behavior of participants, we performed a repeated-measures ANOVA comparing oculomotor suppression effects using factors salience (high vs. low salience) and phase (phase 1 vs. phase 2). There was a main effect of salience, with a larger oculomotor suppression effect for high- (7.7%) than low- (4.7%) salience distractors, *F*(1,23) = 4.82, *p* = .039, *adj. η*_*p*_^*2*^ = .14. There was a main effect of phase with smaller oculomotor suppression effects in phase 2 (5%) than phase 1 (7.4%), *F*(1,23) = 6.4, *p* = .019, *adj. η*_*p*_^*2*^ = .18. Critically, there was no interaction between salience and phase, *F*(1,23) = 0.22, *p* = .64, *adj. η*_*p*_^*2*^ =  − .03. These results suggest that despite smaller oculomotor suppression effects in phase 2, the lack of an interaction between salience and phase indicates participants did not change their suppressive behavior between phases. Thus, we conclude that participants were better able to suppress the high- than low-salience distractors throughout both phases of the experiment.

##### **Saccadic latency**

**Fig. 3:**
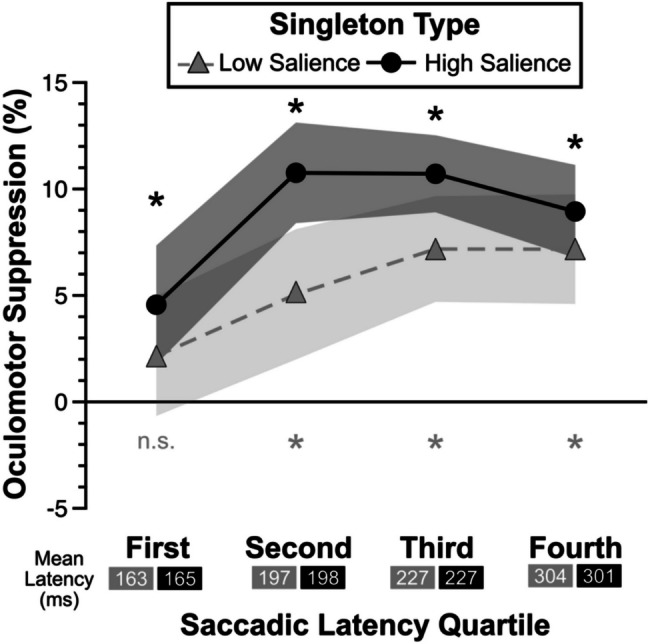
Oculomotor suppression effects as a function saccadic latency quartile for low- and high-salience singletons. Mean saccadic latency for each quartile (low salience/high salience) are provided below the x-axis labels. Error bars represent within-subject 95% confidence intervals. Statistical tests compare each oculomotor suppression effect against zero. *p < .05

To rule out a potential rapid disengagement mechanism (Theeuwes, 2021, 2023) driving the oculomotor suppression effects, specifically the fastest saccades which might be driven more by bottom-up saliency than strategic control (Oor et al., 2023; Zhang et al., 2025b), we examined the oculomotor suppression effects as a function of saccadic latency (Stilwell et al., 2023; van Zoest et al., 2004; Zhang & Gaspelin, 2024). We reasoned that if there was evidence of rapid disengagement, it should emerge earlier during the fastest first saccades given that perceptual saliency can drive the fastest eye movements. To test whether the fastest first saccades resulted in greater oculomotor suppression by the high- than low-salience distractors, we performed post-hoc analyses by deriving oculomotor suppression effects (first fixations on the singleton distractor minus first fixations on the average nonsingleton distractor), binning these effects across four quartiles of saccadic latency (Stilwell et al., 2023), and then performing a repeated-measures ANOVA with factors of salience (low vs. high salience) and quartile (from the fastest 25% of saccades to the slowest 25% of saccades) (Fig. 3). There was a main effect of salience with greater oculomotor suppression for the high- (8.8%) than the low-salience (5.5%) distractors, *F*(1,31) = 11.66, *p* = .002, *adj. η*_*p*_^*2*^ = .25. There was a main effect of quartile, with weaker oculomotor suppression effects during the fastest saccades (3.4%) than any other quartile (8%, 9%, 8.1% for the remaining quartiles, respectively), *F*(3,93) = 8.21, *p* < .001, *adj. η*_*p*_^*2*^ = .18. However, there was no interaction between salience and quartile, *F*(2.04,63.37) = 1.06, *p* = .36, *adj. η*_*p*_^*2*^ = .00, suggesting that the stronger oculomotor suppression effects for the high- than low-salience distractors did not change as a function of how fast the first saccade was deployed (Stilwell et al., 2023; Zhang & Gaspelin, 2024).

##### **Grip choice**

**Fig. 4:**
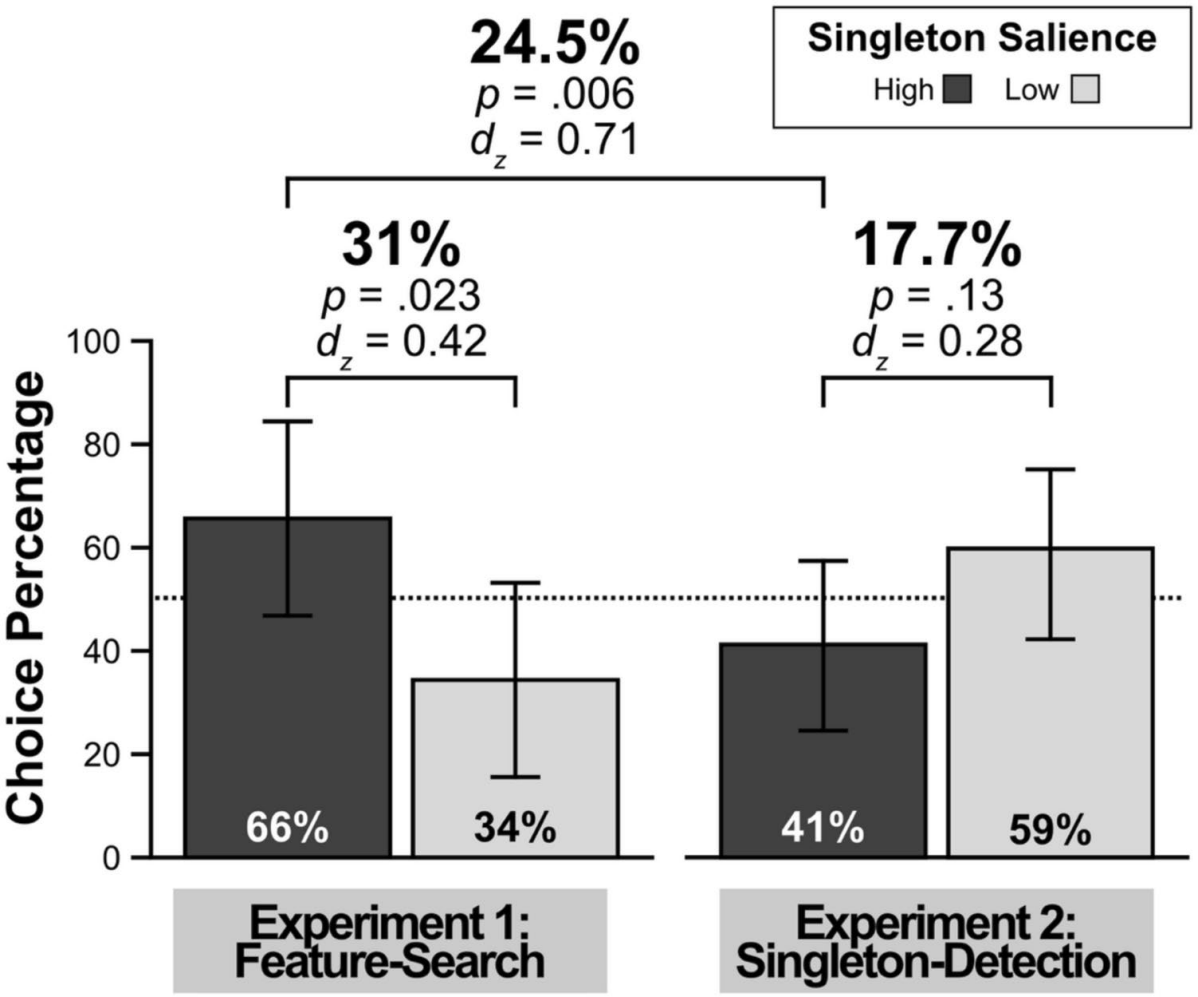
Grip choice percentages for experiments 1 and 2. Choice percentage reflects the percentage of blocks (out of six) participants chose each singleton color. The dotted line depicts chance level choice of 50%. All error bars represent within-subject, 95% confidence intervals (Cousineau, 2005; Morey, 2008)

Participants had the option of choosing to retain or switch the distractor color from the previous block at the start of each experimental block. To switch the distractor color, participants had to squeeze the grip device to their maximum calibrated force. Any percentage of force less than their calibrated maximum would retain the singleton color from the previous block. Thus, the choice of singleton distractor color was effectively a two-alternative forced choice task. Participants had six opportunities to switch the color; therefore the percentage of choice for each singleton color was the number of blocks each color was selected divided by six (Fig. 4). If participants did not have a preference for either singleton color, they should choose the colors equally often (i.e., 50% choice percentage). Given six opportunities to choose the distractor color, chance performance would mean that participants selected each distractor color on average 3 times. Participants were significantly more likely to exert physical effort to select high-salience singleton blocks (65.6%, which was significantly higher than chance) than low-salience singleton blocks (34.4%, which was significantly lower than chance), *t*(31) = 2.4, *p* = .023, *d*_*z*_ = 0.42. Furthermore, of the 32 participants, 19 had a preference for the high-salience color as defined by a greater number of blocks where they chose the high- than low-salience distractor color, whereas nine participants had a preference for the low-salience distractor color, a difference that is signficant via a binomial test, *p* = .044. The remaining four participants had no preference. These results suggest that participants were more willing to exert physical effort to perform searches with the high- than low-salience distractors, which aligns with their greater oculomotor suppression for the high- than low-salience distractors.

## Discussion

In experiment 1, we aimed to replicate the pattern of greater oculomotor suppression for high- than low-salience singleton distractors from Stilwell et al. (2023). Participants suppressed both levels of salient color singleton distractors, with a larger magnitude of oculomotor suppresion for the high- than the low-salience distractors, consistent with Stilwell et al. (2023) (see also Zhang & Gaspelin, 2024). Having established a differential effect of oculomotor behavior based on singleton salience, we next sought to test whether participants would be more willing to exert physical effort to select the color singleton they were more efficient at suppressing. The results of the grip task show that participants were more willing to exert physical effort to experience blocks containing the high- than the low-salience distractors, suggesting that they prefered the distractors that they were able to suppress more robustly.

However, participants may have shown a greater preference for the high-salience singletons for any number of idiosyncratic reasons unrelated to their oculomotor suppression behaviors. For instance, the high-salience singletons are by definition more salient and thus might have been preferred simply because of the strength of their salience signals. To rule out reasons for the high-salience preference that are unrelated to signal suppression, in experiment 2 we adopted a search task that promotes singleton-detection mode, which is known to result in oculomotor capture instead of suppression (Gaspelin et al., 2017). If participants are no longer able to suppress the salient distractors and are similarly distracted by both high- and low-salience items, then they should also show no preference between conditions of distractor salience. If participants exhibit a general preference for high-salience distractors that is unrelated to the efficiency of signal suppression, then the preference for the high-salience singletons should remain in experiment 2, because the salience of the singleton distractors was the same as experiment 1.

## Experiment 2

### Method

#### Participants

A new naïve sample of 32 students from Texas A&M University participated for course credit after obtaining written informed consent (25 women, 7 men; mean age = 19.3 years). All participants were English-speaking and reported normal or corrected-to-normal visual acuity and normal color vision. All procedures were approved by the Texas A&M University Institutional Review Board and were conducted in accordance with the principles expressed in the Declaration of Helinski.

#### Apparatus, design, and procedure

All aspects of the experimental setup, design, and procedure were identical to experiment 1 except for the nature of the search task and stimuli used in the search displays.

#### Search stimuli

As depicted in Fig. 1A, participants searched for the unique shape target which varied randomly from trial-to-trial among homogenously shaped nontargets (i.e., a diamond target among circle nontargets or a circle target among diamond nontargets). All other aspects of the stimuli were identical to those used in experiment 1.

#### Data analysis

The first block of each phase was excluded as practice. Trials with an RT less than 200 ms or greater than 2,000 ms (3.5% of trials in phase 1, 1.1% of trials in phase 2) were removed from analysis. Furthermore, trials with abnormal saccadic latencies less than 50 ms or greater than 1,000 ms (1.5% of trials in phase 1, 0.8% of trials in phase 2) and trials where participants did not move their eyes from central fixation (0.4% of trials in phase 1, 1.4% of trials in phase 2) were removed. Additionally, trials containing an incorrect response (3.0% of trials in phase 1, 2.5% of trials in phase 2) were excluded from all analyses. These trimming procedures resulted in a total of 7.5% and 4.2% of trials excluded from phases 1 and 2, respectively.

### Results and discussion

#### Phase 1: Attentional capture task

##### **Manual responses**

Manual RTs were slower on singleton-present trials than singleton-absent trials (a singleton-presence cost) for both conditions of salience, see Table 2. One-sample *t* tests indicated that the singleton-presence costs for both the high-salience (117 ms), *t*(31) = 11.17, *p* < .001, *d* = 1.97, and the low-salience distractors (91 ms), *t*(31) = 7.67, *p* < .001, *d* = 1.36, were statistically significant. Contrary to the results of experiment 1, a paired-samples *t* test indicated that the singleton-presence costs were larger for the high-salience than the low-salience distractors, *t*(31) = 2.19, *p* = .036, *d*_*z*_ = 0.39, suggesting that the high-salience distractors captured attention more so than the low-salience distractors. Mean error rates were low and showed neither singleton-presence benefits nor costs (*p*’s > .14). Together, these results suggest that participants were captured, as indicated by mean RTs, by the high-salience distractors more than the low-salience distractors, which is the opposite pattern of results of experiment 1.

**Table 2:** Experiment 2: Mean response time (in ms) and mean error rate (in %) by singleton presence and singleton salience

Mean RTs (ms)					
	Phase 1: Attentional Capture Task			Phase 2: Grip Task	
*Singleton salience*		Absent	Present	Absent	Present
Low		972 (111)	1062 (130)	851 (85)	920 (96)
High		969 (132)	1086 (163)	871 (125)	927 (126)
**Mean error rates (%)**					
	Phase 1: Attentional Capture Task			Phase 2: Grip Task	
*Singleton salience*		Absent	Present	Absent	Present
Low		2.4 (2.6)	2.9 (1.9)	2.8 (3.1)	2.2 (2.1)
High		2.1 (2.6)	2.7 (1.8)	2.4 (2.3)	3.0 (2.3)

Standard deviations are provided in parentheses

##### **First saccade destination**

**Fig. 5:**
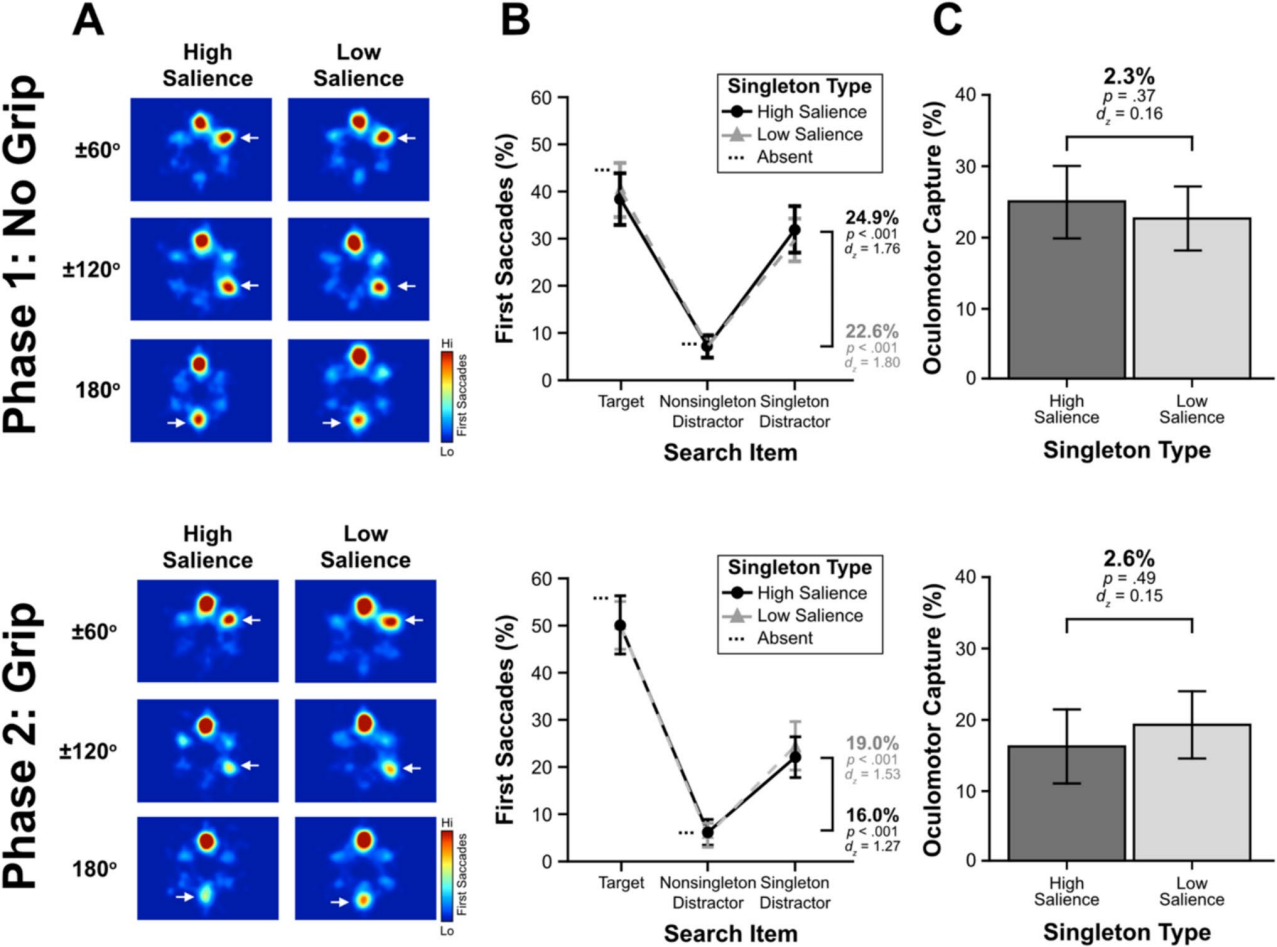
Saccade destination results for experiment 2. Phase 1 results are presented in the top panel and phase 2 results are presented in the bottom panel. (**A**) Heat maps depicting the first saccades as a function of singleton salience and angular distance between the target and the singleton distractor. The heat maps were generated and normalized with the target appearing at the top position and the white arrow depicting the singleton distractor’s location. (**B**) The percentage of first saccades to each search item as a function of singleton salience. (**C**) Oculomotor capture effects (singleton distractor—average nonsingleton) as a function of singleton salience. All error bars represent within-subject, 95% confidence intervals (Cousineau, 2005; Morey, 2008)

The primary aim of phase 1 was to entrain attentional capture to establish the opposite pattern of eye movement behaviors from Experiment 1. Figure 5A depicts heat maps for the first saccade as a function of singleton salience and the angular distance. As shown, participants devoted the majority of their first eye movements toward either the target or the singleton distractor. These first eye movements to the singleton distractor did not appear to change as a function of the distractor’s salience. To assess this pattern statistically, we compared the percentage of first saccade destinations as a function of the search item fixated and singleton salience (Fig. 5B). As in experiment 1, the nonsingleton represents the per-item average for all the nonsingleton distractors. Oculomotor capture is defined as the difference between first saccades landing on the singleton distractor and the average nonsingleton distractor. These oculomotor capture effects are depicted in Fig. 5C, where a positive value indicates the salient distractor captured attention. The high-salience distractors (24.9%), *t*(31) = 9.95, *p* < .001, *d* = 1.76, and the low-salience distractors (22.6%), *t*(31) = 10.20, *p* < .001, *d* = 1.8, clearly captured attention. Importantly, the magnitude of capture did not differ between the high- and low-salience distractors, *t*(31) = 0.92, *p* = .37, *d*_*z*_ = 0.16, suggesting participants were similarly overtly captured by both types of singleton. These results established that participants showed no differential oculomotor behavior toward either level of salience, which contrasts with the pattern of oculomotor behavior of increased suppression for the high- than low-salience distractors in experiment 1.

##### Saccadic latency

**Fig. 6:**
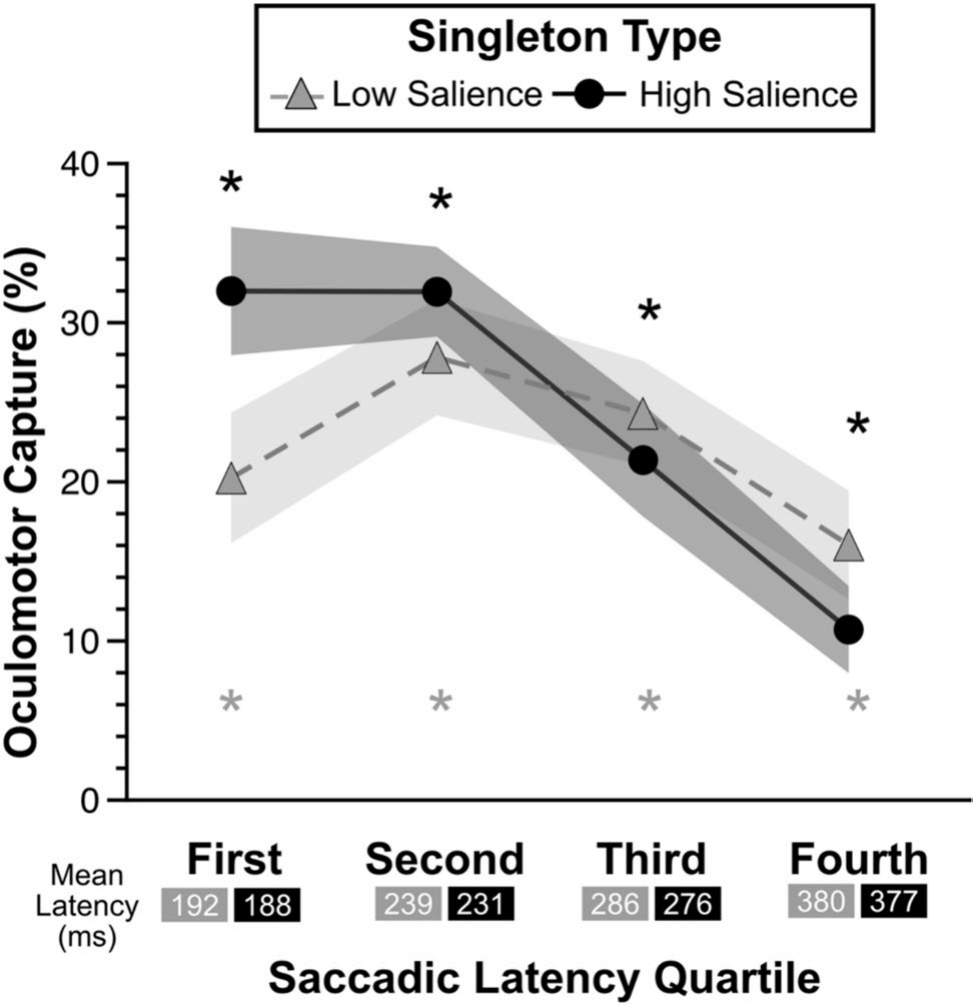
Oculomotor capture effects as a function saccadic latency quartile for low- and high-salience singletons. Mean saccadic latency for each quartile (low salience/high salience) are provided below the x-axis labels. Error bars represent within-subject 95% confidence intervals. Statistical tests compare each oculomotor capture effect against zero. *p < .05

One potentially surprising result is that increasing the salience of the singleton distractor in experiment 2 did not result in greater oculomotor capture by the more salient singleton, although we did observe more robust capture as indexed by mean RTs (Forschack et al., 2023; Hauck et al., 2023; Wang & Theeuwes, 2020). Participants showed numerically larger oculomotor capture effects for the high- than the low-salience distractors in the first phase of experiment 2. To determine whether there was any evidence of stronger oculomotor capture by the high-salience singletons, we examined the oculomotor capture effects as a function of saccadic latency (Stilwell et al., 2023; van Zoest et al., 2004; Zhang & Gaspelin, 2024). We reasoned that if there was any evidence of increased oculomotor capture by the high-salience singletons, it should emerge earlier during the fastest first eye movements given that perceptual saliency can drive the fastest eye movements (Oor et al., 2023; Zhang et al., 2025b). As in experiment 1, we again performed post-hoc analyses by deriving oculomotor capture effects (first fixations on the singleton distractor minus first fixations on the average nonsingleton distractor), binning these effects across four quartiles of saccadic latency (Stilwell et al., 2023), and then performing a repeated-measures ANOVA with factors of salience (low vs. high salience) and quartile (from the fastest 25% of saccades to the slowest 25% of saccades) (Fig. 6). There was no main effect of salience, *F*(1,31) = 0.58, *p* = .45, *adj. η*_*p*_^*2*^ =  − .01. There was a main effect of quartile that followed a U-shape with oculomotor capture effects increasing from the fastest quartile (26.3%) to the second fastest quartile (30.1%), then decreasing for the third fastest (23.0%) and slowest quartiles (13.6%), *F*(1.53,47.37) = 5.54, *p* = .012, *adj. η*_*p*_^*2*^ = .12. However, unlike experiment 1, there was a significant interaction between salience and quartile, *F*(3,93) = 4.98, *p* = .003, *adj. η*_*p*_^*2*^ = .11.

To parse this interaction, we compared oculomotor capture effects between high- and low-salience for each saccadic latency bin. For the fastest quartile of first saccades (*M*_*low*_ = 192 ms; *M*_*high*_ = 188 ms), the high-salience distractors had larger oculomotor capture effects (33.3%) than the low-salience distractors (22.3%), *t*(31) = 3.19, *p* = .003, *d*_*z*_ = 0.56. For the slowest quartile of first saccades (*M*_*low*_ = 380 ms; *M*_*high*_ = 377 ms), the reverse was true: high-salience distractors had smaller oculumotor capture effects (10.8%) than the low-salience distractors (17.4%), *t*(31) = 2.82, *p* = .008, *d*_*z*_ = 0.5. There were no differences in oculomotor capture effects for either of the other two latency quartiles (*p*’s > .41). These results suggest that when participants made relatively fast first saccades (~ 190 ms), they were likely guided by salience, leading to robust capture by the more salient singleton distractor. However, with more time to allow for covert shifts of attention to select and then rapidly disengege from the distractor (Theeuwes et al., 2000) during the slowest first saccades (~ 380 ms), the high-salience distractors were less likely to capture overt attention. This pattern of early capture driven by salience can help to explain the numerical trends toward larger oculomotor capture by the high- than low-salience distractors in experiment 2, given that roughly 25% of first eye movements were more likely to select the high-salience distractor. This latency-based effect could also help explain the difference in RT-based capture between the high- and low-salience distractors. Regardless, these salience-driven effects of the high-salience distractor can explain why participants were numerically less likely to choose the high-salience distractors in experiment 2, which is in line with our hypothesis that participants exert physical effort to match the condition where they perform more efficient visual search.

#### Phase 2: Grip task

##### Manual responses

The magnitude of singleton-presence costs from phase 1 were reduced in phase 2. One-sample *t* tests indicated that the singleton-presence costs for both the high-salience (56 ms), *t*(24) = 4.66, *p* < .001, *d* = 0.93, and the low-salience distractors (69 ms), *t*(29) = 7.42, *p* < .001, *d* = 1.38, remained statistically significant. However, in contrast to phase 1, the difference in singleton-presence costs between the high- and low-salience singletons was in the opposite direction but not statistically reliable, with larger singleton-presence costs for the low- than high-salience distractors, *t*(21) = 1.93, *p* = .068, *d*_*z*_ = 0.41. We compared the singleton-presence costs between phases (phase 1 vs. phase 2) and salience (high vs. low) by using a repeated-measures ANOVA. There was a main effect of phase, with larger singleton-presence costs in phase 1 (101 ms) than in phase 2 (63 ms), *F*(1,21) = 15.07, *p* < .001, *adj. η*_*p*_^*2*^ = .39. There was no main effect of salience, *F*(1,21) = 0.23, *p* = .63, *adj. η*_*p*_^*2*^ =  − .04. The interaction between phase and salience was trending but not significant, *F*(1,21) = 3.57, *p* = .073, *adj. η*_*p*_^*2*^ = .11. Follow-up paired-samples *t* tests revealed that the singleton-presence costs for the high-salience singletons were reduced from phase 1 (117 ms) to phase 2 (56 ms), *t*(24) = 4.2, *p* < .001, *d*_*z*_ = 0.84, but the singleton-presence costs for the low-salience singletons were numerically reduced but did not differ significantly between phases 1 (91 ms) and 2 (69 ms), *t*(28) = 1.94, *p* = .062, *d*_*z*_ = 0.36. These results are consistent with greater suppression of the high- than low-salience distractors over time by reducing the potency of distraction via experience, which could be owing to second-order suppression (Drisdelle et al., 2025; Gaspelin & Luck, 2018b; Ma & Abrams, 2023b; Won et al., 2019). Mean error rates were low overall and did not differ by conditions of salience (*p*’s > . 26).

##### **First saccade destination**

The oculomotor capture effects from phase 1 persisted into phase 2 as evident in Fig. 5 (bottom panel). Comparing oculomotor capture effects, the high-salience distractors (16.0%), *t*(24) = 6.34, *p* < .001, *d* = 1.27, and the low-salience distractors (19.0%), *t*(28) = 8.25, *p* < .001, *d* = 1.53, clearly continued to capture attention. The magnitude of capture did not differ significantly between the high- than low-salience distractors, *t*(21) = 0.70, *p* = .49, *d*_*z*_ = 0.15, replicating the pattern found in phase 1. These results suggest that participants’ oculomotor behaviors did not differ between the levels of salience, as they were overtly captured by both types of singleton distractor.

##### Grip Choice

Consistent with experiment 1, in experiment 2, participants had six opportunities to switch the color; therefore the percentage of choice for each singleton color was the number of blocks each color was selected divided by six (Fig. 4). Unlike experiment 1, in experiment 2 in which participants showed no difference in oculomotor capture between the levels of salience, they also showed no difference in color preference. Participants were similarly likely to exert physical effort to select both the high-salience singleton blocks (41.2%) and low-salience singleton blocks (58.9%), *t*(31) = 1.55, *p* = .13, *d*_*z*_ = 0.27, which was no different from chance. If anything, the preference was in the opposite direction as in experiment 1: a numerically larger preference for the low- than high-salience distractors. Further, of the 32 participants, eight had a preference for the high-salience color as defined by a greater number of blocks where they chose the high- than low-salience distractor color, whereas 14 participants had a preference for the low-salience distractor color. The remaining 10 participants had no preference either way. These results suggest that participants were just as likely to choose each distractor color based on its salience and align with the oculomotor capture effects in that there were no differences between oculomotor capture as a function of salience.

#### Combined analysis relating preference to oculomotor behavior

**Fig. 7:**
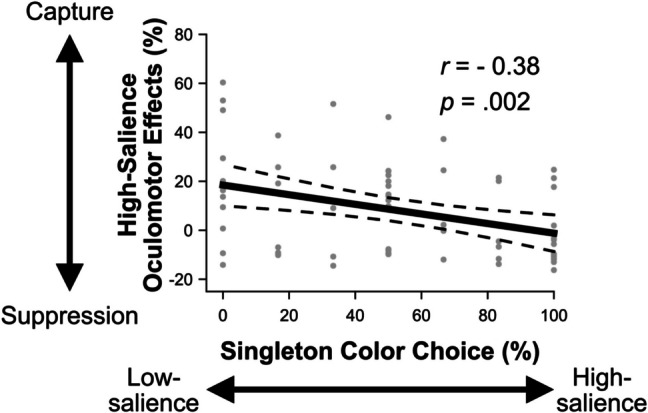
Correlation results for the combined data from experiments 1 and 2. The y-axis depicts the magnitude of oculomotor effects for the high-salience singleton condition in phase 1. Oculomotor effects are the difference between first saccades directed toward the singleton distractor minus the average nonsingleton distractor. Positive values reflect oculomotor capture while negative values reflect oculomotor suppression. The x-axis depicts choice preference for either condition of salience defined as the percentage of blocks participants exerted maximum physical effort to select that color. Higher percentages reflect a greater preference for the high-salience singleton color while lower percentages reflect a greater preference for the low-salience singleton color. The gray circles represent individual participants’ values, and the dashed lines depict 95% confidence intervals for the correlation

We correlated the magnitude of participants’ oculomotor effects for the high-salience singleton distractors during phase 1 with their choice preference for the high-salience singleton blocks in phase 2 by combining the data from both experiments. The oculomotor effects were defined as the difference between first saccades directed toward the singleton distractor minus the average nonsingleton distractor, with a positive value indicating oculomotor capture and a negative value indicating oculomotor suppression. The percentage of blocks chosen for each level of salience was defined relative to the high-salience singleton, such that larger percentages would reflect a greater preference for the high-salience singleton and lower percentages would reflect a greater preference for the low-salience singleton. As evident in Fig. 7, the magnitude of oculomotor capture was negatively correlated with singleton-preference, *r* =  − .38, *p* = .002. Participants prefered to exert physical effort for the high-salience distractor blocks when they had shown stronger magnitudes of oculomotor suppression for the high-salience distractors during phase 1. This pattern suggests that the choice preference for the high-salience distractors reflects their oculomotor suppression.

#### Summary of experiment 2

In experiment 2, we aimed to establish a condition where participants would be overtly captured by the salient color singletons instead of being able to suppress them, to test whether the preference for the high-salience distractors in experiment 1 was unrelated to signal suppression. The oculomotor results were clear in that participants were overtly captured by singleton distractors regardless of their salience. If the preference for the high-salience distractors was due to any number of idiosyncratic reasons unrelated to signal suppression, then when participants no longer showed a difference in oculomotor effects between conditions of salience they should also show no preference for either condition of salience. In experiment 2, participants were no more willing to exert physical effort for high- than low-salience distractors; if anything, they were more likely to work for the low-salience distractors, which could be partially explained by the greater capture by high- than low-salience distractors on mean RTs. Further, the magnitude of oculomotor suppression correlated with preference for the high-salience singletons such that the more efficiently participants suppressed the high-salience singletons the more likely they were to exert physical effort to search through displays containing them.

## General discussion

In the current study, we aimed to test whether suppressing highly salient singleton distractors was more or less effortful than suppressing less salient distractors. To test this question, participants completed an initial phase of a visual search task designed to entrain suppression of high- and low-salience distractors via a color contrast manipulation (Stilwell et al., 2023). Immidiately following the first phase, participants then completed a second phase where they were given the choice of singleton distractor color at the beginning of each block via physical effort exertion (i.e., squeezing a hand dynamometer). We hypothesized that if suppressing the high-salience distractors made visual search easier and less effortful via reduced competition from the distractor in perceptual systems, then participants should be more willing to exert physical effort to choose the search task that contained the high-salience distractor. In contrast, if the recruitment of suppressive mechanisms of attentional control, which are more robustly recruited by high-salience distractors, is itself an efforful process, then we should expect the opposite pattern of results. In experiment 1, we observed greater oculomotor suppression for the high- than low-salience distractors (Stilwell et al., 2023; Zhang & Gaspelin, 2024). Furthermore, participants were more willing to exert physical effort to search through blocks containing the high- than the low-salience distractors. We argue that these results reflect participants’ perceived effort: Participants were more willing to exert physical effort to search with trials containing the high-salience distractor which they more efficiently suppressed.

In experiment 2, we ruled out such suppression-independent possibilities by switching the search task where the high-salience distractors should, if anything, result in less efficient search than the low-salience distractors. Participants were similarly captured by both the high- and low-salience distractors and as a result, showed no preference for either singleton type using the grip manipulation. Comparing the strength of oculomotor suppression and singleton preference across both experiments, the more efficiently participants suppressed the high-salience singletons, the more willing they were to exert physical effort to search through displays containing those distractors. Together, these results suggest that participants perceived suppressing the high-salience distractors as less effortful than suppressing the low-salience distractors.

The pattern of greater suppression for the high- than low-salience distractors replicates previous demonstrations of the same result (Stilwell et al., 2023; Zhang & Gaspelin, 2024) which further lends support for the notion that increasing the salience of the singleton distractor does not neccesitate stronger attentional capture (Wang & Theeuwes, 2020). Instead, the results are consistent with the signal suppression hypothesis whereby highly salient singleton distractors can be suppressed (Gaspelin & Luck, 2018a; Gaspelin et al., 2017; Stilwell et al., 2022, 2024). However, there does seem to be a potential limit to how salient a distractor can be before it can no longer be suppressed. For example, dynamic stimuli, such as abrupt onsets (Zhang et al., 2025c), might have a potent enough signal to overpower suppression (Adams & Gaspelin, 2024; Jonides & Yantis, 1988) unless they contain a suppressible feature, such as color (Adams et al., 2022). The current study cannot speak to whether color singletons are unique compared with dynamic stimili, but the current set of results does support the idea that increasing the salience of a static distractor (e.g., via color contrast) can lead to greater suppression and a correspondingly stronger preference to search under such conditions.

The willingness to exert physical effort to select the condition that promotes more efficient visual search replicates recent findings (Anderson et al., 2025). For example, participants are more willing to exert physical effort (i.e., via squeeing a hand dynamometer) to search through displays containing fewer items (Anderson & Lee, 2023), to search via feature-search mode instead of singleton-detection mode (Lee et al., 2024), or to reduce the frequency of distractor items (Anderson, 2024a). The current study replicates and extends these results by demonstrating that participants are willing to exert effort to search through displays containing a more salient distractor which they are more efficient at suppressing. This suggests that there is some utility in the ability to suppress more salient distractors and participants seem to perceive their increased suppression ability as worth some measure of physical effort exertion in exchange for more opportunities to exercise this ability. We suspect that there are at least two candidate mechanisms for why the ability to suppress a more salient distractor might be perceived as less cognitively effortful.

One plausible mechanism could involve competition from the distractor in the formation of and/or the readout from the attentional priority map (Anderson, 2021b). According to the signal suppression hypothesis (Gaspelin & Luck, 2018c), the salient signal generated by the singleton distractor can be proactively suppressed, preventing the signal from accumulating enough evidence to trigger a shift of attention toward that item. Although not formally articulated in the signal suppression hypothesis, it stands to reason that a more salient signal would trigger the suppressive process to begin and/or complete sooner and/or be more likely to complete within a set timeframe. There is some neurobiological evidence consistent with the idea that more salient singletons start the suppressive process earlier. For example, the early P_D_ component (Gaspelin et al., 2023) and *Ppc* ERP component (Barras & Kerzel, 2016; Corriveau et al., 2012) both seem to be larger in amplitude and earlier in time when singleton distractors are more salient (Drisdelle & Eimer, 2023; Kerzel & Huynh Cong, 2022). Additional evidence comes from visual brain networks in nonhuman primates, including single unit recordings in the frontal eye field (FEF) and laterial intraparietal (LIP) area, where there are distinct populations of neurons that encode visual salience with stronger correlations to distractor suppression behavior in FEF (Sapountzis et al., 2025; Thompson & Bichot, 2005). Together, these findings point to early deviations in visual processing based on visual salience, lending support to a signal suppressive process triggering sooner for more salient stimuli. This is turn could lead to the salient signal being suppressed sooner and more efficiently, preventing downstream effects of attentional selection of the salient distractor. Initiating the process earlier would effectively save the visual system the need to trigger later, and perhaps more demanding, reactive inhibitory processes (Braver, 2012; Geng, 2014).

A second plausible mechanism could involve the engagement of attentional control and the maintainence of an attentional control state (Anderson, 2024c; Luck et al., 2021). In the context of the current study, the ability to maintain an attentional control state that upweights the target while downweighting the salient distractor may be more effective the more dissimilar these two stimuli are, specifically when participants are searching using a feature-search mode (as in experiment 1). In other words, the ability to maintain the attentional control setting to prioritize a specific shape (e.g., experiment 1) in the presence of a high-salience distractor might be more efficient and require less cognitive demand than the same search in the presence of a low-salience distractor owing to the target-distractor dissimilarity. The more distinct the salient distractor, the more readily it can be distinguished from the target and each subject to selective information processing. The closer the target and nontargets are in feature space, the longer it takes to reject search items that are not the target and the more likely the attention system is to confuse the target with a nontarget and thereby select the nontarget (Duncan & Humphreys, 1989). We remain agnostic to which of these two mechanisms (if not both, as they are not mutually exclusive and are to some degree interdependent) drives the preference for searching among high-salience distractors. Future research is needed to more definitely support either or both mechanisms.

One limitation of the current study concerns the manipulation of preference for the singleton distractors. We used a more conservative manipulation of physical effort exertion in which participants were never *required* to exert physical effort. Participants in the current study were always given the option of squeezing the hand dynamometer if they wanted to, but they did not need to ever grip the device to perform the task. Given this conservative approach, one limitation of the current results is that the preference participants showed might not truly reflect a trade-off between exerting physical effort for a less demanding search task. In other words, the perceived cost to the participant to squeeze the hand dynanometer at the beginning of each block (six times total) might have been low enough that participants were not sufficiently motivated to exert physical effort. However, this same conservative approach could be interpreted in the opposite direction: Despite a potentially weak motivation to exert physical effort, participants were nonetheless motivated enough to exert effort in exchange for displays containing the high-salience singleton distractors. Participants were never required to exert physical effort; therefore their choice to do so provides more compelling support for the notion that they perceived the high-salience distractors as easier to suppress. Future studies could raise the costs associated with participants’ choices, creating a stronger incentive to exert more physical effort: (“If you really want the high-salience singletons, you are going to have to really work for it!”).

## Conclusion

The current study aimed to test whether more salient distractors are easier to suppress than less salient ones. We replicated the pattern that increasing the salience of a singleton distractor leads to greater suppression (Stilwell et al., 2023). After establishing that participants suppress the high-salience distractors more efficiently, we then gave them the option to exert physical effort in exchange for the ability to suppress those distractors. Participants were more willing to exert physical effort in exchange for suppressing the high- than low-salience distractors. These findings have important implications for the cognitive demands associated with salient distractor suppression. The more salient a singleton distractor becomes, the easier it seems to be to suppress and to identify the target of a visual search.

## Data Availability

All data, analysis scripts, and stimulus presentation programs in this article are available on the Open Science Framework at https://osf.io/3zegr.

## Footnotes

1 In the oddball detection task, participants were presented brief search displays and were tasked with detecting whether a color singleton was present or absent. The duration of the displays prior to a post-mask was varied following the logic that more salient color singletons should require briefer exposure durations to be reliably detected than less salient singletons. The high-contrast colors were reliably detected with briefer durations than the low-contrast colors, suggesting they were perceptually more salient (Stilwell et al., 2023). We used the same high- and low-contrast colors in the current study as Stilwell et al. (2023).

2 If we had changed the target/nonsingleton colors between phases, we would be unsure whether the grip choice would reflect distractor-related or target/nonsingleton-related processes. Since the distractor colors were the only colors to change throughout the experiment by their condition of salience, we could be more confident that any change in grip choice as a function of the salience of the distractors would reflect distractor- not target-related processing.

3 It is a bit peculiar that the RT differences do not map on directly to our primary dependent measure (i.e., our oculomotor measures) (Drisdelle & Eimer, 2023; Stilwell et al., 2023). We suspect that the RT differences reflect any and all processes during the search task (e.g., the initial attentional guidance, selection, response selection and execution); whereas the first saccade data reflects a purer measure of the first shift of attention and selection, which should be uncontaminated by later, post-selection/guidance processes (Hollingworth & Bahle, 2020). We did observe manual RT-based singleton-presence benefits (experiment 1) and costs (experiment 2) for both distractors; we just failed to observe a difference between conditions of salience. Stilwell et al. (2023), which this study was a close replication of, also found the same pattern for RTs. This may be related to recent findings that eye movement indicators of distractor suppression are considerably more reliable than RT differences in this context ( Kim et al., 2024).

4 It is important to note that in phase 2 participants chose which distractor color would appear in each block of trials; therefore, some participants did not have trials for a given salience condition if they did not choose to have those blocks of trials. This makes it difficult to compare between the conditions of salience given the two conditions were not balanced across participants.
